# Sclerosing Epithelioid Fibrosarcoma of the Bone: A Case Report of High Resistance to Chemotherapy and a Survey of the Literature

**DOI:** 10.1155/2010/431627

**Published:** 2010-04-12

**Authors:** Thomas G. P. Grunewald, Irene von Luettichau, Gregor Weirich, Angela Wawer, Uta Behrends, Peter M. Prodinger, Gernot Jundt, Stefan S. Bielack, Reiner Gradinger, Stefan Burdach

**Affiliations:** ^1^Department of Pediatrics and Pediatric Oncology Center (POC), Klinikum rechts der Isar, Technische Universität München, Kölner Platz 1, 80804 Munich, Germany; ^2^Institute of Pathology, Technische Universität München, Ismaninger Straße 22, 81675 Munich, Germany; ^3^Department for Orthopedics and Trauma Surgery, Klinikum rechts der Isar, Technische Universität München, Ismaninger Straße 22, 81675 Munich, Germany; ^4^Institute of Pathology, University of Basel, Schönbeinstraße 40, 4003 Basel, Switzerland; ^5^Klinik für Kinder- und Jugendmedizin, Pädiatrie 5 (Onkologie, Hämatologie, Immunologie), Klinikum Stuttgart, Olgahospital, Bismarckstraße 8, 70176 Stuttgart, Germany

## Abstract

Sclerosing epithelioid fibrosarcoma (SEF) is a rare soft tissue sarcoma mostly occurring in extraosseous sites. SEF represents a clinically challenging entity especially because no standardized treatment regimens are available. Intraosseous localization is an additional challenge with respect to the therapeutical approach. We report on a 16-year-old patient with SEF of the right proximal tibia. The patient underwent standardized neoadjuvant chemotherapy analogous to the EURAMOS-1 protocol for the treatment of osteosarcoma followed by tumor resection and endoprosthetic reconstruction. Histopathological analysis of the resected tumor showed >90% vital tumor cells suggesting no response to chemotherapy. Therefore, therapy was reassigned to the CWS 2002 High-Risk protocol for the treatment of soft tissue sarcoma. To date (22 months after diagnosis), there is no evidence of relapse or metastasis. Our data suggest that SEF may be resistant to a chemotherapy regimen containing Cisplatin, Doxorubicin, and Methotrexate, which should be considered in planning treatment for patients with SEF.

## 1. Introduction

Sclerosing epithelioid fibrosarcoma (SEF) is an uncommon fibrosarcoma of intermediate-grade malignancy and has lately been recognized as a distinct clinical entity [1]. SEF is a malignancy of the older adult with a mean patient age of 47 years (range 14–87 years) [2], but onset in adolescence has also been reported [1, 3]. Only 10% of patients are younger than 20 years at time of diagnosis. The distribution between sexes seems to be equal [4]. SEFs mainly present as tumors of the lower extremities (39%), followed by the trunk (21%) and upper extremities (14.5%), but also rare locations like pituitary gland, intraspinal or base of penis have been described [1–8]. Typically SEF is complicated by high frequencies of local tumor relapse and distant metastases (30%–40%) [2–4, 9–11]. Metastases mostly occur in lung (70%), bone (41%) and soft tissue [2–4] with a median interval of 7.7 years from diagnosis to first apparent metastasis [1], but according to a recent meta-analysis up to 27% of patients display distant metastases already at time of diagnosis [2]. In addition, SEF shares the potential for lymph node metastasis with other epithelioid malignant soft tissue tumors such as epithelioid sarcoma [12, 13] and epithelioid malignant peripheral nerve sheath tumor [14]. Follow-up data indicate that SEF is an aggressive tumor with mortality rates ranging between 25% and 57% [1, 4].

Together with low-grade fibromyxoid sarcoma (FMS) and hyalinizing spindle cell tumor with giant rosettes [15], SEF belongs to the rare family of fibrosing fibrosarcomas only a few pathologists have encountered [16, 17]. Each of these tumors has distinct clinical features, but they share common histological components suggesting a close relationship [15, 18–21]. Histologically, SEF predominantly consists of epithelioid cells arranged in strands, nests, and/or sheets, and set in a fibrotic and extensively hyalinized stroma [4, 15]. The presence of epithelioid cells often leads to diagnostic confusion with metastatic carcinoma and malignant lymphoma [1]. Further diagnostic difficulties arise from the large paucicellular fibrous zones and focal myxoid areas, features also seen in low-grade FMS [4]. Ultrastructurally, cells of SEF resemble fibroblasts due to abundant rough endoplasmic reticulum [22]. Thus, pathologists worldwide agree to the complexity of the histological picture, which often leads to equivocal and delayed diagnosis [2–4, 7, 8, 14, 18, 23–29].

The only consistent immunohistochemical finding is a strong and diffuse reactivity for vimentin, while almost all other markers are negative [3, 4]. Peculiar to this tumor is a subset of cases that stain for EMA [1, 3, 8], a finding that may be related to the tumor's epithelioid phenotype. Some rare cases express NSE [1, 29] or S-100 protein [1, 20, 27]; however, staining is weak or merely focal. In their study of 25 cases, Meis-Kindblom et al. concluded that SEF is a relatively low-grade fibrosarcoma that is fully malignant despite the presence of histologically benign appearing foci [1]. In a recent case study metastases of a SEF presented with a much higher proliferative activity indicated by 60% of the tumor cells staining positive for the proliferation marker Ki-67 versus 7%-8% of the primary tumor [28]. In other cases even lower initial Ki-67-positivity was observed (1%–6%) [31]. These findings suggest that SEF can progress to much more aggressive phenotypes [28]. Moreover, Jiao et al. could detect strong immunoreactivity with murine double minute 2 (MDM2) in the absence of p53 mutations in one case, pointing to a possible role of MDM2 overexpression in tumorigenesis of SEF [22].

## 2. The Case

We report on a 16-year-old female who presented with a four-week history of persistent and progressive load-dependent pain focused on the right proximal tibia. On physical examination, no signs of inflammation or other malfunctions could be seen. Conventional X-rays of the right tibia revealed a 4 × 4 cm osteolytic cavity, which was presumptively diagnosed as a benign lesion. Thus, open biopsy was carried out, and pathological differential diagnosis of an *ossifying fibroma* or *desmoplastic fibroma* was reported. Intermittently, the pain relieved and the patient was discharged. Four weeks later, local pain was recurring and an X-ray control showed progression of the osteolytic lesion. Hence, curettage and reconstruction with autologous bone graft of the iliac bone was performed. Again, tissue specimens, now described as *non-ossifying fibroma*, presented as a benign process in the histological workup. Decreasing pain and present stability of the leg allowed the patient's discharge. Three months later the patient was reassessed by open biopsy due to recurrent and increasing pain as well as swelling of the proximal right tibia. This time, tissue specimens were analyzed by independent local and reference pathologists who finally established the diagnosis of SEF of the bone. As seen in other cases of SEF the resected tumor only stained positive for vimentin in immunohistochemistry. Routine staging was negative for metastases (CT-scan of the lung and 18-FDG-PET/CT scan). Clinically, only one single enlarged lymph node at the right outer thoracic wall was detected, which, however, showed no tumor cell infiltration on resection biopsy.

Taking into consideration the bone association of the tumor and the lack of a standardized treatment regimen, the local interdisciplinary tumor board decided to treat the patient following the EURAMOS-1 (European-American-Osteosarcoma-1) protocol designed for the treatment of osteosarcoma. The EURAMOS-1 treatment plan consists of chemotherapy elements with Doxorubicin, Cisplatin, and high-dose Methotrexate [32], and several case reports describe treatment of patients with SEF with these drugs [2]. Yet, clinical follow-up and documentation of these cases is not conclusive enough to predict clinical benefit of this therapy [2].

**Figure 1:**
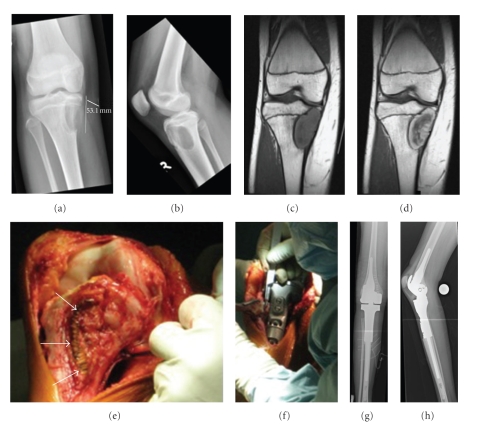
*Pre- and postoperative radiological imaging of the right leg and in situ pictures:* (a and b) preoperative conventional X-rays of the right knee. The tumor shows an eccentric osteolytic lesion located at the epimetaphyseal proximal tibia, not respecting the epiphyseal plate. The medial cortical bone is completely destroyed and the formation of partially calcified periosteal lining suggests a Lodwick-type 1C lesion. (c) MRI scan demonstrating a T1 isointense, T2 hypointense tumor formation. A similar zonal architecture with a large central core of very low signal intensity and a peripheral rim of intermediate to high signal intensity on T1- and T2-weighted spin-echo pulse sequences was observed by Christensen et al. [30]. (d) MRI scan showing high uptake of Gadolinium predominantly at the tumor's periphery. A thin layer of sclerotic bone separates the tumor from circumjacent marrow edema. (e and f) Intraoperative images during tumor resection and implantation of the endoprosthesis. Resection of the tumor was performed in no-touch technique. The scar of the previous open biopsy (white arrows) as well as the former access path remained on the resected bone. Reconstruction was accomplished by implanting a partially custom-made tumor endoprosthesis in combination with linked knee replacement. (g and h) Postoperative radiographs of the right leg show the endoprosthesis in proper position.

**Figure 2:**
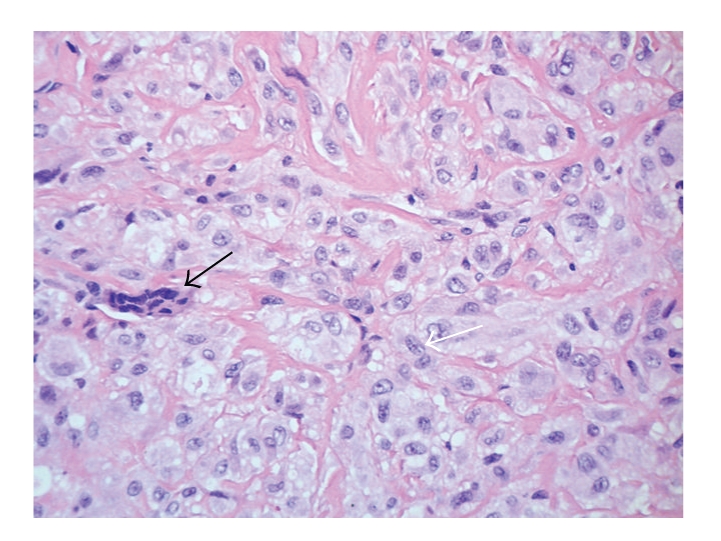
*H&E staining of sclerosing epithelioid fibrosarcoma* after treatment with neoadjuvant chemotherapy (magnification ×400). The tumor is composed of few scattered polynuclear giant cells (black arrow) and groups of cells, which are rich in cytoplasm and show pale, vesicular, and irregular nuclei without any substantial mitotic activity (white arrow). The tumor cells are embedded in a collagen-rich extracellular matrix and display no signs of regression.

**Figure 3:**
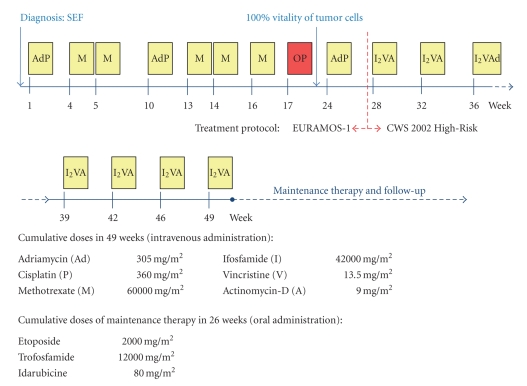
*Schematic illustration of the treatment regimen* including cumulative dosages of the chemotherapeutic drugs. Each chemotherapy cycle of the main therapy is depicted as a yellow box, and the week when the cycle started is shown below. The time point of surgery and implantation of endoprosthesis is indicated with a red box (OP). The regimens of the maintenance therapy and follow-up are not displayed in detail.

After two courses of Cisplatin/Doxorubicin and five courses of high-dose Methotrexate our patient underwent tumor resection and endoprosthetic reconstruction (Figure 1). The resected tumor specimen revealed marginal safety distance at the resection boarders. Moreover, the tumor showed no signs of regression in the resection specimen (grade of regression VI according to Salzer-Kuntschik [33]) (Figure 2). These findings prompted the decision to change the chemotherapy regimen and to introduce a different set of agents. Thus, at this point, in accordance with the expert panel at the biannual meeting of the GPOH, we started to treat the patient according to the CWS 2002 High-Risk protocol (German Cooperative Soft Tissue Sarcoma Study; Cooperative Weichteilsarkom Studie) designed for the treatment of soft tissue sarcoma [34]. The adjuvant chemotherapy was now based on Ifosfamide, Vincristine, and Actinomycin-D. Altogether the patient received seven courses of chemotherapy according to CWS 2002 High-Risk protocol [32, 34] and eight courses of an orally administered maintenance therapy consisting of Idarubicine, Etoposide, and Trofosfamide (overview in Figure 3). To date, 22 months after diagnosis of SEF, the patient is well and attended regularly in our outpatient clinic. There is no evidence of relapse and/or metastasis so far.

## 3. Discussion

In general, SEF appears to be a slowly growing tumor often present for several months or years before diagnosis. In most cases of SEF, it took 33 months from the first onset of symptoms to correct diagnosis [2]. The delayed diagnosis (4 months) in our patient once again emphasizes the difficulty arising from the inconclusive clinical, radiological, and histopathological presentation of this tumor. Although the tumor macroscopically has a circumscript appearance, it routinely infiltrates soft tissues, including periosteum, and can even invade bone [1–4, 35]. To our knowledge this is the third case of a SEF primary arising from the bone [35–37], which led to the initial clinical diagnosis of *ossifying fibroma* in our patient. Indeed, osseous differentiation of SEF has been described in the literature and was seen in the first biopsy, but was absent in specimens of the second biopsy [3]. Moreover, infiltration of the adjacent bone has previously been reported [1–4]. Although histomorphology of SEF suggests being low-grade, it clinically presents as a high-grade tumor [2, 28]. SEF belongs to the family of fibrosing fibrosarcomas and appears to be the most malignant variant of this family of low-grade fibrosarcomas [2]. However, the high mortality rate observed in patients with SEF may also be due to the lack of experience of most of the physicians in how to treat patients with SEF potentially leading to inadequate therapy and unfavorable outcome [2].

## 4. Conclusion

Due to the rareness of this tumor, there are no established treatment regimens. So far, patients have been treated with amputation, wide excision, radio- and chemotherapy, or various combinations thereof [2]. Our experience showed that Doxorubicin, Methotrexate, and Cisplatin had no significant effect on tumor vitality. This information and the fact that SEF clinically presents with features of high-grade tumors should be considered when deciding on the treatment. The role of systemic therapy, however, still remains unclear. Future follow-up of our patient will demonstrate whether chemotherapy according to the CWS-protocol and tumor resection can prevent the development of metastasis and relapse.

##  Consent

Written informed consent was obtained from the patient for publication of this case report and any accompanying images. A copy of the written consent is available for review by the Editor-in-Chief of this journal.
